# Malignant struma ovarii: Case report of an unusual ovarian tumor with CT imaging

**DOI:** 10.1016/j.radcr.2022.02.067

**Published:** 2022-03-22

**Authors:** Atrikha Rahma, Lies Mardiyana, Dyah Fauziah

**Affiliations:** a Department of Radiology, Faculty of Medicine Universitas Airlangga, Dr Soetomo General Academic Hospital, Surabaya, Indonesia; b Department of Anatomic Pathology, Faculty of Medicine Universitas Airlangga, Dr Soetomo General Academic Hospital, Surabaya, Indonesia

**Keywords:** CT Imaging, Malignant Struma Ovarii, Ovarian Teratoma

## Abstract

Struma ovarii is categorised as a monodermal type of mature teratoma and consists primarily of thyroid tissue. It is an uncommon ovarian tumor, with non–specific clinical, and imaging findings. The majority of struma ovarii are benign and are typically associated with mature cystic teratomas. A small proportion of struma ovarii may undergo malignant transformation, with papillary carcinoma the most common type of malignancy. The criteria used to identify a malignant change in struma ovarii are identical to those used to evaluate the thyroid gland. Nevertheless, due to the rarity of struma ovarii, imaging diagnostic criteria, and subsequent management are not clearly defined. This case report describes a 41-year-old female patient who presented with rapid abdominal enlargement over a period of 1 month, accompanied by elevation of the tumor marker CA-125. Based on these clinically findings, ovarian cancer was suspected. The patient underwent a total abdominal hysterectomy with bilateral salpingo-oophorectomy. Histopathology results revealed a malignant struma ovarii.

## Introduction

Teratoma comprises 15%-20% of ovarian tumors. Ovarian teratoma is a germinal cell tumor arising from foetal yolk sac germinal cells. Based on the WHO classification, ovarian teratomas can be divided into 3 groups: mature, immature, and monodermal. Because these teratomas are germinal cell tumors, they are typically differentiated from the embryonic germ layer into 3 components: ectoderm, endoderm, and mesoderm. As a result, these tumors demonstrate a wide variety of morphologies. A monodermal teratoma is defined as an ovarian teratoma that contains predominantly or solely one type of tissue [1]. A diagnosis of struma ovarii is made when thyroid tissue comprises more than 50% of the tumor tissue. Struma ovarii represents 1% of all ovarian tumors and 2.7% of all dermoid tumors [2]. Preoperative diagnosis is difficult, due to a lack of distinctive clinical and imaging features, and the rate of preoperative misdiagnosis is high. Because it is an uncommon type of tumor, there are no specific clinical, radiological, or serum markers that distinguish struma ovarii in the absence of thyroid hormone abnormalities. Thus, a definitive diagnosis is obtained by histopathological examination. In addition, preoperative radiological findings are crucial for determining appropriate management [3]. In this case report, we present a patient with malignant struma ovarii and describe the identification of the tumor's features with computed tomography (CT) imaging, based on a thorough review of the literature.

## Case report

**Fig. 1:**
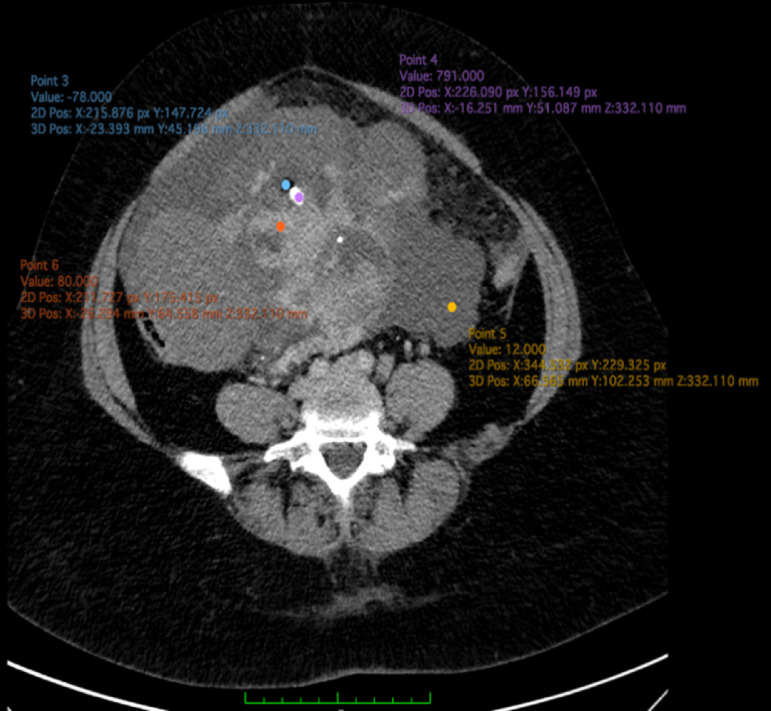
Axial CT view in venous phase, revealing a dominant cystic mass (12 HU, yellow dot) located on the right ovary, with papillary projections, a solid component (80 HU, orange dot), calcification (791 HU, purple dot), and a fat component (-78 HU, blue dot). The mass is multilobulated, with a smooth margin measuring approximately 10.0 × 15.6 × 22.6 cm. Ascites can also be observed in the abdominal and pelvic cavity (asterisk) (Color version of the figure is available online.)

**Fig. 2:**
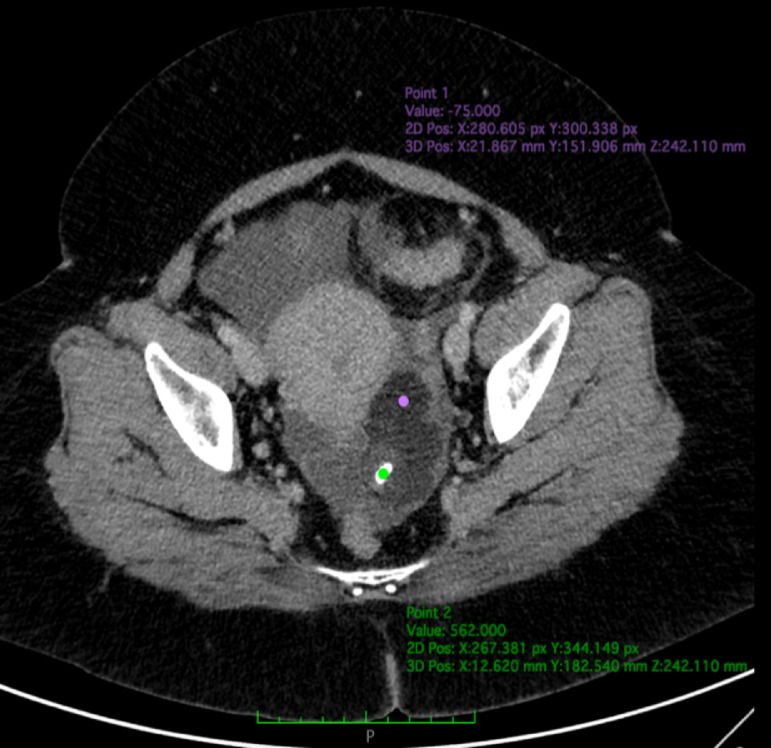
Axial CT view in venous phase, showing a mass with a fat-dominant (-75 HU, purple dot) component, minimal solid component, and calcification (562 HU, green dot), measuring 5.4 × 3.6 × 7.0 cm on the left ovary. Ascites is apparent in the abdominal and pelvic cavities (asterisk) (Color version of the figure is available online.)

**Fig. 3:**
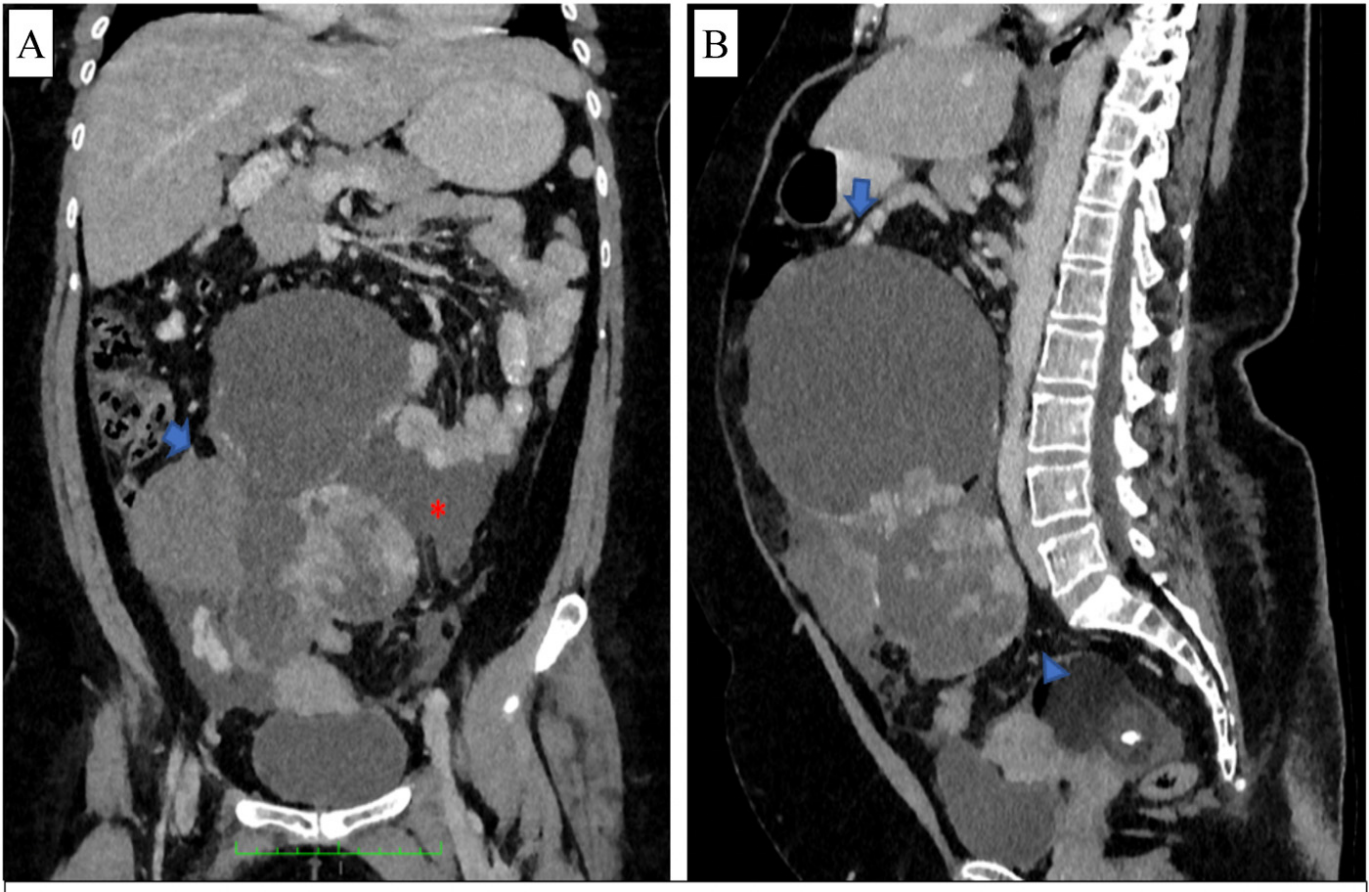
Abdominal multi-slice CT (MSCT): (A) coronal and (B) sagittal views in the venous phase, demonstrating adnexal masses on the right ovary (arrow) and left ovary, which have cystic, fat, solid, and calcification components. The solid component shows contrast enhancement, and ascites in the abdominal and pelvic cavities (asterisk).

**Fig. 4:**
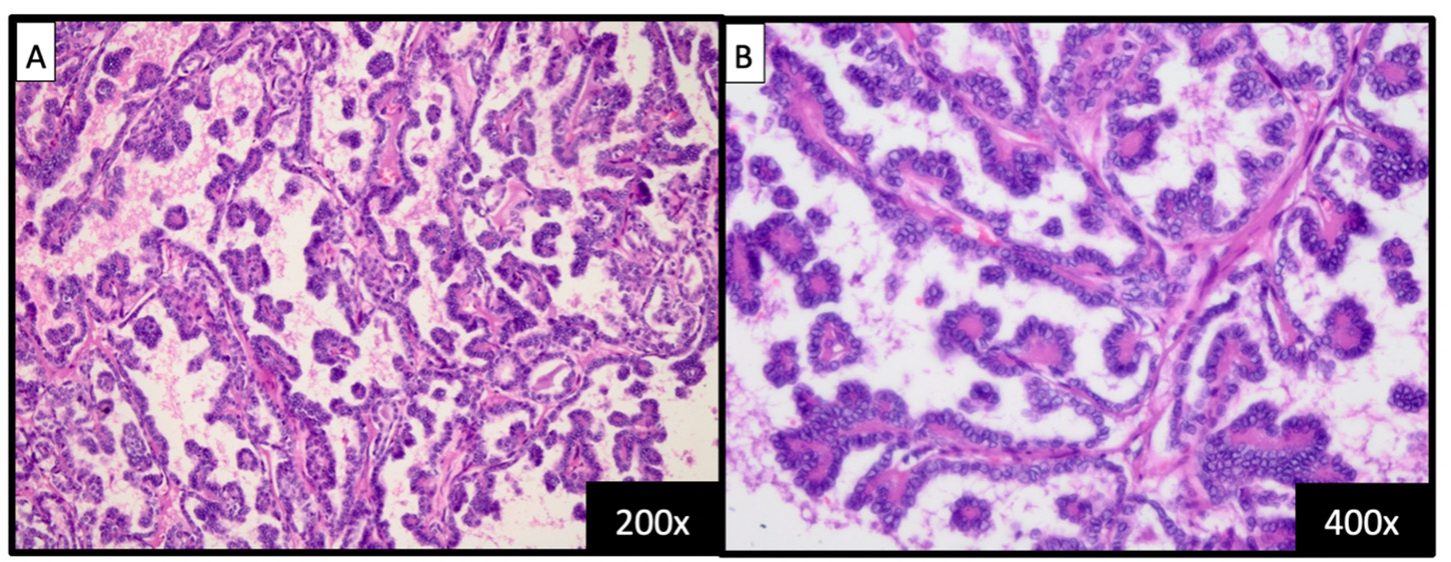
(A) Pathology examination revealed a papillary tumor with a fibrovascular stalk (H&E, 200x). (B) The papillary structures were lined with epithelial cells displaying round-shapes with a ground glass appearance and nuclear groove (H&E, 400x).

A 41-year-old female patient presented with rapid abdominal enlargement for a period of 1 month. Serum levels of tumor marker CA-125 were elevated at 604.22 U/mL (normal: less than 30-35 U/mL), but serum carcinoembryonic antigen (CEA) levels were considered normal. Ascites was observed in the abdominal and pelvic cavities. The clinician suspected ovarian cancer; however, paracentesis revealed benign peritoneal ascites. CT imaging showed a dominant cystic mass with solid and papillary projection components, calcification, and a fat component, with a multilobulated, smooth margin measuring approximately 10.0 × 15.6 × 22.6 cm from the right ovary (Fig. 1, Fig. 2, Fig. 3), and a dominant fat mass and solid component with calcification, measuring 5.4 × 3.6 × 7.0 m from the left ovary (Fig. 1, Fig. 2, Fig. 3). Moreover, the mass showed contrast enhancement in the solid component. The patient underwent a total abdominal hysterectomy with bilateral salpingo-oophorectomy. Histopathological examination revealed a malignant struma ovarii of the right ovary (Fig. 4), whereas the left ovary contained a benign teratoma.

## Discussion

Struma ovarii is a germ cell tumor of the ovary. It is comprised of more than 50% thyroid tissue and can be differentiated from a mature teratoma, which contains only a small component (less than 50%) of benign thyroid tissue. Despite the high thyroid tissue content of such tumors, thyrotoxicosis occurs in only 5% of all struma ovarii cases [4]. Struma ovarii typically arises unilaterally, with 5% of cases occurring bilaterally [5]. Struma ovarii may mimic the clinical symptoms of ovarian malignancy, presenting with ascites, a complex ovarian cyst, and an elevation of CA-125 [2]. Struma ovarii occurs primarily in women aged 30-50 years and may be accompanied by contralateral mature teratoma ovarii and cystadenoma [6].

On CT imaging, struma ovarii generally reveals a complex cystic appearance with non–specific findings. The presence of invasive mass growth, a large solid component, and an irregular border on the mass wall or within the mass are typical CT findings in ovarian teratoma with malignant transformation. It is difficult to differentiate between benign and malignant struma ovarii without extracapsular extension, however, due to the presence of ascites, which is common in malignant masses and can also occur in benign struma ovarii [7]. The overall appearance of the struma consists of multiple cystic and solid areas. Moreover, the gross pathologic appearance of the struma is characterised by a solid area [3]. The high attenuation and calcification observed in the solid areas of these tumors are characteristics of struma ovarii that are detectable with CT imaging. Struma ovarii can also be identified by the presence of a high-density cyst, and CT imaging may show solid, cystic, or cystic-solid components. In the present case, cystic-solid components were found to be the most frequent type; however, the least frequent type also occurs in solid components through CT scan examination. Unlike the most common types of teratoma, struma ovarii does not display lipid material on CT imaging. However, the presence of lipid material and fatty tissue could be indicative of the presence of a dermoid cyst, which is associated with struma ovarii. Understanding these findings is essential for a reliable diagnosis of this type of tumor [3]. Marked enhancement of locally thickened walls and thick septations are indicative of struma ovarii when imaging is carried out after administration of an intravenous contrast agent. The solid components that microscopically correspond to thyroid tissue show strong enhancement as well. This is likely due to the increased vasculature and fibrous content of the thyroid tissue and stroma in the solid part of the struma ovarii tumor. Some experts have suggested that these findings are due to thyroglobulin and thyroid hormones in the follicular thyroid tissue that significantly attenuates X-rays. Additionally, struma ovarii in the presence of ascites is considered complicated [3]. Ascites occurs in 17% of cases; however, the ascitic fluid rarely contains tumor cells [8]. Malignant struma ovarii typically has a tumor size ranging from 3-20 cm [4].

When an ovarian tumor has both solid and cystic components, further investigation is needed, prior to surgery, to determine whether it is likely benign or malignant. Other useful criteria for evaluating malignant ovarii are mass size (greater than 4 cm), the thickness of septa and wall (greater than 3 mm), and the presence of an internal structure. The latter includes various degrees of solid components, papillary projections, nodularity, necrosis, and haemorrhage. The imaging findings of benign and malignant ovarian lesions may overlap significantly. As the imaging of enhancement patterns improves, however, it will increase the accuracy of differentiating malignant, and benign lesions [9].

## Conclusion

Struma ovarii is an uncommon and generally benign tumor. Nevertheless, a small proportion of struma ovarii may undergo malignant transformation. CT scan with contrast administration can determine whether a struma ovarii is likely benign or malignant by a thorough examination of the character of the mass, enhancement pattern, the thickness of septa and wall, and presence of an internal structure. Pathology examination of the surgical specimen is mandatory to determine malignancy in struma ovarii.

## Patient consent

Informed consent obtained for publication of a case report. Written informed consent was obtained from the patient for the publication of this case report.
